# Long-term follow-up after ureteral reimplantation in children: a 12-year analysis

**DOI:** 10.1007/s00383-025-05992-1

**Published:** 2025-03-15

**Authors:** T. Gerwinn, M. Zellner, A. Prouza, U. Kennedy, M. Horst, L. Mazzone

**Affiliations:** 1 Division of Pediatric Urology, University Children’s Hospital Zurich, Zurich, Switzerland; 2 Children’s Research Center, University Children’s Hospital Zurich, Zurich, Switzerland; 3 Department of Diagnostic Imaging, University Children’s Hospital Zurich, Zurich, Switzerland

**Keywords:** Ureteral reimplantation, Vesicoureteral reflux, Obstruction, Reflux nephropathy, Children

## Abstract

**Purpose:**

To evaluate the incidence of late surgical complications and long-term kidney outcomes following ureteral reimplantation (UR) for high-grade vesicoureteral reflux (VUR) and primary obstructed megaureter (POM), assessing the necessity of routine long-term follow-up.

**Methods:**

A retrospective review was conducted of 135 children who underwent UR between 2006 and 2013, with at least 10 years of follow-up. Primary outcomes included postoperative ureterovesical junction obstruction (pUVJO) and febrile urinary tract infections (UTIs). Secondary outcomes assessed kidney function and morphology (renal ultrasound changes, elevated BUN/creatinine, and arterial hypertension).

**Results:**

Fifty-three renal units in 34 children met the inclusion criteria. pUVJO occurred in 7.5% of units, exclusively after Politano–Leadbetter (P–L) reimplantation, and within eight months postoperatively. Nine children experienced febrile UTIs, primarily within the first postoperative year. One child with reflux nephropathy (RN) developed chronic kidney disease 12 years later, and another developed RN without impaired function. No hypertension was observed in the cohort.

**Conclusion:**

Children with uneventful recoveries and no pUVJO in the first year have a low risk of late complications, suggesting limited benefit from routine long-term follow-up. Higher risk groups, including those undergoing P–L reimplantation, girls, and those with preoperative RN, may warrant extended follow-up.

## Introduction

Ureteral reimplantation (UR) is a well-established procedure in the management of vesicoureteral reflux (VUR) and primary obstructive megaureter (POM) in children, providing success rates around 95–98% [1]. However, postoperative follow-up practices vary substantially, influenced by institutional protocols and individual surgeon preferences. The American Urological Association's (AUA) recommendation for a postoperative ultrasound follow-up recognizes the marginal risk associated with postoperative ureterovesical junction obstruction (pUVJO). But it does not specify the frequency and extent of ultrasound follow-up [2]. Similarly, the European Association of Urology (EAU) recommends postoperative follow-up until after puberty, but without specifying diagnostic parameters or ultrasound intervals [3].

Indeed, a postoperative ultrasound at the three-month mark, devoid of pUVJO has been suggested as a robust predictor of an uneventful postoperative course [3]. As a result, some have advocated that asymptomatic children may not benefit from extended, routine follow-up [4–7]. However, these conclusions arise from relatively short-term follow-up analysis and rely primarily on imaging studies, neglecting the broader spectrum of potential long-term complications and changes after UR. For instance, recurrent febrile UTIs may serve as indicators for postoperative VUR, and the exceptionally rare late presenting pUVJO, manifesting years after an otherwise uneventful postoperative period, remains widely unaddressed [8, 9]. Additionally, complications linked to the underlying congenital anomaly of the kidney and urinary tract (CAKUT), such as reflux nephropathy (RN), arterial hypertension, and chronic kidney disease (CKD), should not be disregarded. The existing body of evidence, particularly with respect to thorough, longitudinal long-term follow-up after UR, remains notably limited, leaving a conspicuous void in the understanding of the evolving health of these children [4–7].

Recognizing the existing limitations in follow-up duration and study design of prior investigations, this study seeks to address the potential emergence of late-presenting complications and changes post UR. Our aim is to conduct a thorough assessment of our study population to identify vulnerable individuals who may benefit from standardized, longitudinal long-term follow-up.

## Methods

**Study approval**: The study received approval from the Cantonal Ethics Committee of Zurich (BASEC-No. 2022-02253).

**Population**: We conducted a retrospective review of 135 medical records of children who underwent UR at our institution between January 2006 and June 2013. Inclusion required a minimum 10-year postoperative follow-up after UR for primary VUR with breakthrough febrile UTIs, secondary VUR after obstructive ureterocele incision, or POM. We enrolled cases with unilateral and bilateral UR for single system kidneys as well as duplex kidneys. Each side was counted as a renal unit. Common sheath UR for duplex kidneys with complete ureteral duplication were counted as one renal unit. We excluded subjects with urogenital, anorectal or bladder abnormalities and those with an underlying malignant or non-malignant systemic disease. Seventy-seven children had insufficient follow-up duration and were not included in this study.

**Surgical technique**: Ureteral reimplantation was performed using either the Cohen or Politano–Leadbetter technique, with the choice made intraoperatively at the surgeon's discretion. In duplex systems, ureters were reimplanted within a common sheath. Ureteral tapering was performed as needed, typically for massively dilated, non-peristaltic ureters.

**Standard postoperative follow-up**: We offer clinical, blood pressure and ultrasound follow-up visits scheduled at 3, 12, 24 months, and subsequently every four years until adolescence, typically around 16 years of age, where the need for transitioning to adult urology practice will be determined. BUN/creatinine measurement is not routinely ordered but is performed in cases of bilateral renal changes, inadequate renal growth, or a history of abnormal results in previous exams. Our institutional protocol mandates the continuation of antibiotic prophylaxis for three months following surgery. Postoperative VCUG is not performed routinely but is reserved for patients with recurrent UTIs after surgery. Bladder retraining strategies are not routinely included in follow-up but may be offered if concomitant lower urinary tract dysfunction is diagnosed.

**Primary outcome**: Surgical complications, including pUVJO (due to stenosed intravesical channel or retrovesical ureteral kinking), and febrile UTIs as indicators for postoperative VUR. We documented the time of diagnosis and the technique used for reoperation.

**Secondary outcome**: Indicators of kidney impairment, such as parenchymal changes detected on ultrasound, arterial hypertension, or elevated levels of blood urea nitrogen (BUN) and creatinine.

**Data collection**: Demographic data, including gender, age at surgery and duration of follow-up were collected. We recorded the presence of single or duplicated kidney, VUR grade and laterality, SFU grade and laterality, POM, preoperative RN, the indication for surgery, previous urogenital interventions or surgeries, the intraoperative details including the surgical technique employed (Cohen, Politano–Leadbetter, common-sheath UR), the need for ureteric tapering, the insertion of externalized ureteral catheters and details about postoperative antibiotic prophylaxis.

Medical histories were evaluated, categorizing symptomatic individuals as those with a confirmed febrile UTI or afebrile UTI (defined as abnormal urine analysis, a positive urine culture and/or clinical signs suggestive for UTI). Additionally, we considered arterial hypertension, lower urinary tract dysfunction (LUTD) according to the committee of the international Children’s Continence society [10], increased serum creatinine/BUN and estimated glomerular filtration rate (eGFR).

**Ultrasound evaluation**: A senior board-certified pediatric radiologist assessed preoperative ultrasound images, one-year ultrasounds for short-term evaluation, ultrasounds at 5 ± 2 years for mid-term analysis, and those at ≥ 10 years for long-term follow-up. These assessments were performed in accordance with the Society for Fetal Urology's (SFU) grading system [11]. Additionally, parenchymal changes detected on ultrasound, indicative of RN, were documented. These included renal cortical thinning, loss of cortico-medullary differentiation, focal cortical scars, and reduced renal size. For cases with follow-up exceeding ten years, the most recent ultrasound was considered for long-term sonographic follow-up.

**Data analysis**: Due to the limited cohort size, we used descriptive statistics.

## Results

Of the subjects assessed, 53 renal units in 34 children met the inclusion criteria.

Our study cohort consisted of 15 boys and 19 girls, with a mean age at surgery of 2.6 years (range 0.4–7.2 years). The average duration of follow-up was 12.2 years, ranging from 10.0 to 15.2 years. Indication for surgery included primary VUR in 34 units, secondary VUR after ureterocele incision in eight units, and POM in eleven units. Preoperative SFU grade was determined as grade I in 18, grade II in 19, grade III in eight, and grade IV in eight units. One renal unit showed no preoperative dilatation. Preoperative ultrasound revealed parenchymal changes suggestive of RN in three renal units. Procedures included 33 Cohen UR, twelve common sheath Cohen, six P–L, and two common sheath P–L. The study cohort and results are summarized in Table 1.

**Table 1:** ^a^Female: male = 10:2, ^b^mean, ^c^one patient

		Outcome			
		pUVJO	UTI	Reflux nephropathy	Uneventful
Outcome	Total renal units	4	12^a^	4	33
	Time of diagnosis	8.3 m^b^ (7–9 m)	1y^b^ (2w–4y)	3 × pre-OP 1 × 12y post-OP	–
Demographics	Age at surgery	1.2y^b^	2.6y^b^	3.7y^b^	2.4y^b^
	Follow-up	11.7y^b^	13.1y^b^	12.3y^b^	12y^b^
	Duplex kidney	1 (25%)	5 (42%)	1 (25%)	8 (24%)
Indication for surgery	*VUR*	3 (75%)	10 (83%)	4 (100%)	25 (76%)
	Grade I	–	–	–	1
	Grade II	–	3	–	10
	Grade III	1	4	2	4
	Grade IV	–	3	–	9
	Grade V	2	–	2	1
	POM	1 (25%)	2 (17%)	–	8 (24%)
Pre-operative SFU grade	No dilatation	–	1 (8%)	–	–
	Grade I	–	7 (58%)	1 (25%)	10 (30%)
	Grade II	1 (25%)	1 (8%)	3 (75%)	14 (42%)
	Grade III	–	2 (17%)	–	6 (18%)
	Grade IV	3 (75%)	1 (8%)	–	3 (9%)
Previous surgical history	Endoscopic DxHA	2 (50%)	1 (8%)	1 (25%)	2 (6%)
	Ureterocele incision	1 (25%)	4 (33%)	–	3 (9%)
Surgical technique	Cohen	–	7 (58%)	3 (75%)	23 (70%)
	Cohen common sheath	–	5 (42%)	1 (25%)	6 (18%)
	Politano–Leadbetter	3 (75%)	–	–	3 (9%)
	Politano–Leadbetter common sheath	1 (25%)	–	–	1 (3%)
	Ureter tapering	2 (50%)	1 (8%)	–	2 (6%)
	Externalized ureteral catheters	3 (75%)	4 (33%)	–	4 (12%)
Follow-up	Febrile UTI	–	12 (100%)	–	–
	Creatinine/BUN↑	–	–	1^c^	–
	LUTD	1 (25%)	1 (8%)	–	3 (9%)
	Arterial hypertension	–	–	–	–

### Primary outcomes

Postoperative UVJ obstruction was diagnosed in three cases within the first year following P–L reimplantation in four renal units (one bilateral procedure), with an average time of diagnosis at 8.3 months (range seven to nine months) after surgery. No cases of pUVJO were observed after Cohen procedures and none occurred during mid- and long-term follow-up. Three of the four units with obstruction had preoperative grade IV hydronephrosis, one had grade II. All children with pUVJO were asymptomatic, but exhibited persistent or worsening grade III or IV hydronephrosis, compared to the pre-operative ultrasound, at the three-month follow-up (Table 1). The diagnosis of pUVJO was confirmed by prolonged washout or drainage curves and decrease in differential renal function on sequential renal nuclear functional studies (MAG3), guiding indication for re-operation.

Following a more detailed description of the findings: Obstruction in the P–L channel was found during the redo Cohen procedure in a child initially treated for POM with diminished split kidney function.

In another child with secondary grade III VUR following ureterocele incision in a duplicated system's upper pole, common sheath P–L reimplantation led to obstruction caused by a fibrous retrovesical upper pole ureter segment. Re-operation involved a successful laparoscopic ureteroureterostomy, anastomosing the obstructed upper pole ureter to the unobstructed lower pole ureter, leading to full resolution. This child developed constipation-associated LUTD during follow-up.

The final case involved bilateral P-L with excisional ureteral tapering, was done for grade V VUR and recurrent febrile UTIs after failed endoscopic treatment with injection of Dextranomer hyaluronic acid copolymer (DxHA). A bilateral redo Cohen effectively resolved pUVJO, revealing kinking of the retrovesical ureter as the cause of obstructions on both sides. In three of the renal units with pUVJO, ureteral catheters were left in place for one week along with a suprapubic catheter.

Nine children (seven girls and two boys), with twelve renal units (ten units in girls and two in boys), experienced postoperative febrile UTIs (Table 1). Indication for UR was primary VUR and febrile UTIs in six units, VUR after ureterocele incision in four units and worsening obstruction in POM in two units (Table 1). Seven units had preoperative SFU grade I hydronephrosis, two had grade III, and one each had no hydronephrosis, grade II or IV hydronephrosis. Preoperative interventions had been performed in five units, involving four ureterocele incisions and one endoscopic injection of DxHA. Surgical techniques included seven Cohen procedures and five common sheath Cohen for duplicated systems. Ureter tapering was done in one unit, and externalized ureteral catheters were left in place in four units for an average of seven days. Six out of nine children became symptomatic within the first year after UR, including three UTIs despite antibiotic prophylaxis. The remaining three children presented with febrile UTIs four years post-surgery with previously uneventful postoperative courses. Five subjects with postoperative febrile UTIs had duplex kidneys. It is worth noting that all febrile UTIs were isolated events, and further diagnostic workup with VCUG was omitted. One child developed symptoms of LUTD (dysfunctional voiding) during follow-up, successfully treated with pelvic floor exercises and biofeedback. None of the cases with fUTIs had ultrasound evidence of RN until follow-up was concluded.

### Secondary outcomes

Three children, with four renal units, showed signs of RN on ultrasound. Among the four units, three had preoperative SFU grade II hydronephrosis, and one had grade I (Table 1).

One child with a duplex kidney, treated with common sheath Cohen for grade III VUR and recurrent febrile UTIs at 3.3 years, was diagnosed with lower pole RN only during late-term follow-up, twelve years after successful UR, with an uneventful postoperative recovery until then.

The second child, with preoperative RN and a history of recurrent breakthrough UTIs in the setting of grade V VUR, developed mild CKD with an eGFR of 87 ml/min/1.73 m^2^ calculated using the Cockcroft–Gault formula, recorded 12 years after bilateral Cohen procedure at 2.9 years. Previous follow-up visits were uneventful, without febrile UTIs, with normal blood pressure, creatinine and BUN.

The third child with apparent preoperative RN received Cohen reimplantation at age 4.9 years for grade III VUR and a history of multiple febrile UTIs. Postoperative follow-up was uncomplicated, with normal blood pressure measurements, creatinine/BUN levels, and no occurrences of postoperative UTIs. Antibiotic prophylaxis for the individuals with RN was discontinued five months post-surgery on average.

The remaining 33 renal units demonstrated uneventful postoperative courses over a mean follow-up period of twelve years (Table 1). Preoperative hydronephrosis was categorized as SFU grade I in ten cases, grade II in 14 cases, grade III in six cases, and grade IV in three cases. This subgroup of uneventful courses included eight duplex kidneys. Indication for surgery was primary VUR with UTIs in 22 units, secondary VUR in three cases, and eight cases of POM. Preoperative interventions, including ureterocele incision in three cases, endoscopic injection of DxHA in two cases, and two cases of previous uncomplicated upper pole heminephrectomy were documented. Cohen was performed in 23 units, and common sheath Cohen for six units. P–L was done in three cases, and one common sheath P–L in a duplex kidney. Ureter tapering was carried out during two procedures, and externalized ureter catheters were left in place in four units for 1 week. Postoperative antibiotic prophylaxis was administered for an average of five months. Three subjects developed LUTD symptoms (dysfunctional voiding), not associated with UTIs and successfully treated with pelvic floor exercises and biofeedback.

## Discussion

Our study found that pUVJO occurred in 7.5% of the renal units, with diagnoses made within eight months post-surgery. Notably, all cases of pUVJO were exclusively associated with P–L reimplantation and were asymptomatic. Single episodes of postoperative febrile UTIs occurred in a quarter of our study population, predominantly during the first year and in girls. Notably, no recurrent febrile UTIs, indicative for recurrent VUR, were diagnosed. More than a decade after surgery, one child developed CKD, on the basis of preoperatively known RN. During long-term follow-up, one child was diagnosed with RN, yet kidney function remained normal. No study participant developed arterial hypertension.

The recently updated AUA guidelines recommend a renal ultrasound following UR. However, no specific time or frequency were provided due to insufficient data for evidence-based recommendations on post-surgical follow-up [2]. The recommendation emphasizes the potential risk of pUVJO, leading to impaired urinary drainage and subsequent renal damage [2]. The AUA identified an overall pUVJO rate of less than 1% through an analysis of multiple articles, without distinguishing between operative techniques [2]. If hydronephrosis resolves and RN is ruled out, optional annual follow-up is suggested until adolescence, incorporating blood pressure, height, weight, and urinalysis for proteinuria and UTI [2]. The EAU recommends postoperative follow-up until after puberty, but lacks specifics on diagnostic parameters or ultrasound intervals [3].

In our study cohort, pUVJO was found more commonly than reported in the literature. However, it was never diagnosed in the late follow-up, extending beyond one year postoperatively. The uncertainty regarding the precise timing of pUVJO has been discussed in the literature [2]. The onset of pUVJO remains less explored due to its rarity, necessitating multi-institutional studies for sufficient case accumulation and study power. According to the available evidence, diagnosis of pUVJO is typically made between one week to six months after UR. For instance, Falkensammer et al. noted self-limiting pUVJO in 4/126 (3%) cases diagnosed at the 6-month follow-up, remaining clinically asymptomatic with spontaneous resolution of hydronephrosis during continued follow-up [7]. Ellsworth et al. observed pUVJO in 3/108 (3%) cases, requiring cystoscopic JJ-catheter placement within one week post-surgery [5]. Ultrasound findings were normal after JJ-catheter removal one month later [5]. However, rare and historical case reports mention delayed pUVJO occurring years after surgery with uneventful postoperative recovery [8, 9]. Weiss et al. presented two cases of late pUVJO post P–L procedures, attributed to stenosis of the reimplanted intramural ureter. pUVJO was resolved by redo-reimplantation and lysis of fibrotic tissue at the ureteral orifice respectively [8]. A literature review on post-UR hydronephrosis reveals significant variations in the definition of pUVJO, complicating the interpretation and comparison of existing literature on pUVJO after UR [4]. As also demonstrated in our cohort, pUVJO are more frequently reported following the P–L than Cohen intravesical reimplantation. Known drawbacks include the risk of prevesical ureter kinking with subsequent obstruction, while this approach allows for the construction of a longer tunnel, which proves beneficial in severe dilatation [1]. Combined evaluation of three major studies, comprising data from more than 1,200 renal units, reveals an estimated obstruction rate after P–L ranging from 1 to 4% [12–14]. Workarounds to reduce the risk of prehiatal kinking, such as the Mathisen and Psoas hitch techniques, have been described [15–17]. In our cohort, one out of four units with pUVJO was associated with POM. Based on the existing literature, it appears unlikely that POM per se poses an elevated risk for pUVJO. Vereecken et al. found no pUVJO among 52 surgically corrected POMs, including 22 with ureter tapering [18]. William et al. described a cohort of 31 reimplanted POMs with a 90% success rate, and without reporting pUVJO [19]. Gimpel et al. investigated twelve subjects with POM undergoing surgical interventions, utilizing various techniques, and reported just one case of pUVJO requiring reoperation [20]. Taking into account the characteristics of our cohort and the available evidence in the literature, the necessity of regular sonographic follow-up until adolescence for detecting pUVJO becomes questionable, particularly if the renal ultrasound conducted one year after UR reveals no signs of pUVJO, such as progressive or unremitting dilatation.

Nine out of 34 children, equivalent to 26.5%, experienced febrile UTIs postoperatively, with seven out of nine cases occurring in girls. Six children developed symptoms within the first year after surgery. The high rate of postoperative UTIs is in line with studies providing larger case numbers focusing on pyelonephritis-free outcomes after VUR correction [21]. In a retrospective review of over a thousand children, Nelson et al. found that 22% presented with at least one febrile UTI post-VUR correction, occurring on average 8.7 months post-surgery, with 73% of affected individuals being female [21]. Multivariate risk analysis revealed that individuals of female gender, with preoperative VUR grade ≥ III, preoperative breakthrough UTIs, and apparent preoperative RN were more prone to develop postoperative pyelonephritis [21]. Beetz et al. found that 18% of their 158 enrollees, had at least one postoperative febrile UTI in the first ten years post-VUR correction, with the highest probability during the initial postoperative year, especially in females [22].

While RN, CKD and end-stage renal disease can result from recurrent febrile UTIs in VUR, and thus could be preventable by timely surgical correction, they may also be part of CAKUT, predisposing the children for kidney impairment [23]. Despite four units showing RN, none of our enrollees developed arterial hypertension throughout follow-up. Still, blood pressure monitoring is of great importance, as arterial hypertension is common in pediatric and adult subjects with RN [24]. A 15-year follow-up study revealed 13% of pediatric subjects with RN developed arterial hypertension, with bilateral RN linked to earlier hypertension onset (age 22 vs. 30 for unilateral RN) [25].

Due to the enduring risk of children developing RN despite corrected VUR, regular blood pressure and urine protein tests are warranted in primary care, providing benefits for affected children and optimizing healthcare resource allocation.

Our study spanned a substantial duration of twelve years, allowing for a longitudinal follow-up. This extensive timeframe provided us with the opportunity to conduct a comprehensive analysis of long-term surgical outcomes and to monitor the longitudinal development of kidney health within our study cohort. To the best of our knowledge, a longitudinal follow-up after UR of such duration has not been presented in the literature.

This study has several limitations. The small cohort size is a primary concern. 101 of 135 patients had to be excluded, 77 of them due to the requirement of follow-up of at least 10 years. While this ensured robust longitudinal data, it limited the study's scope and generalizability. Additionally, the heterogeneity of underlying pathologies complicates result interpretation, as children with different conditions may respond differently to surgery. Future studies should stratify patients based on their pathology and aim to identify subgroups at higher risk. Reflux nephropathy was diagnosed based on ultrasound imaging, as renal isotope studies were not routinely performed, potentially missing subtle scarring. Lastly, the observed high obstruction rate prompted an internal review, investigating all patients who underwent UR in the last 20 years. This analysis revealed that all cases of pUVJO were captured within this cohort, suggesting selection bias. Our overall pUVJO rate is less than 1% aligning with rates reported in the literature.

## Conclusion

Children who experience uneventful recovery post-UR without signs of pUVJO within the first year demonstrate a low subsequent risk for surgical complications, suggesting limited long-term follow-up necessity.

However, our findings highlight vulnerable groups, including those undergoing the P–L procedure, those with pre-operative RN, and girls, who are more prone to complications and may derive benefit from extended follow-up.

## Data Availability

No datasets were generated or analysed during the current study.

## Abbreviations

AUA: American Urological Association
CKD: Chronic kidney disease
CAKUT: Congenital anomaly of the kidney and urinary tract
DxHA: Dextranomer hyaluronic acid copolymer
eGFR: Estimated glomerular filtration rate
EAU: European Association of Urology
LUTD: Lower urinary tract dysfunction
POM: Primary obstructive megaureter
P–L: Politano–Leadbetter
pUVJO: Postoperative ureterovesical junction obstruction
RN: Reflux nephropathy
SFU: Society for Fetal Urology
UR: Ureteral reimplantation
UTI: Urinary tract infections
VUR: Vesicoureteral reflux
